# Participant Perceptions of a Trauma-Informed Care Planning Intervention: Results from Qualitative Interviews

**DOI:** 10.1093/geroni/igaf122.1487

**Published:** 2025-12-31

**Authors:** Christine Kimpel, Victoria Finamore, Lorely Chavez, Christian Ketel, Kate Clouse

**Affiliations:** The Ohio State University, Columbus, Ohio, United States; Vanderbilt University Medical Center, Nashville, Tennessee, United States; Vanderbilt University, Nashville, Tennessee, United States; The University of Alabama at Birmingham, Birmingham, Alabama, United States; Vanderbilt University, Nashville, Tennessee, United States

## Abstract

Low advance care planning (ACP) participation among subsidized housing residents may result from a higher prevalence of adverse life experiences and traumatic responses to threatening topics, necessitating a trauma-informed approach to foster safety and trust. Using a qualitative descriptive design, we aimed to understand participant perceptions of a trauma-informed advance care planning (TI-ACP) intervention - a one-time, one-to-two-hour conversation guided by a discussion checklist and brief trauma education (symptom recognition and re-traumatization avoidance). In a southeast Nashville subsidized housing community, we conducted one-time, individual, face-to-face, five-to-twenty-minute, semi-structured interviews with adult residents after the intervention. Two coders individually and iteratively coded transcripts, resulting in a hierarchical codebook. Final themes emerged from an iterative thematic analysis process by inductively grouping codes and quotes and deductive application of the SAMHSA trauma-informed care principles. Participants had a median age of 60 (IQR=56-64), and the majority identified as men (52%) and African American/Black (62%) (n = 29). Three themes (ten sub-themes) emerged, including people (participant psychological readiness and capacity for understanding; interventionist’s down-to-earth demeanor and professionalism); process (nonjudgmental, therapeutic, and direct communication; personalized style; and private and comfortable setting), and products (trust: social connection; safety: feelings of ease and relaxation; and raised awareness and empowerment). Our novel approach provided a participant-informed model of how TI-ACP is operationalized. Clinicians and community health workers should promote safety and trust for low SES patients by reinforcing privacy, assessing psychological readiness, and responding with compassion and friendliness. Recommendations for research, policy, and practice are provided.

